# Laminin 521 Stabilizes the Pluripotency Expression Pattern of Human Embryonic Stem Cells Initially Derived on Feeder Cells

**DOI:** 10.1155/2018/7127042

**Published:** 2018-02-18

**Authors:** Halima Albalushi, Magdalena Kurek, Leif Karlsson, Luise Landreh, Kristín Rós Kjartansdóttir, Olle Söder, Outi Hovatta, Jan-Bernd Stukenborg

**Affiliations:** ^1^Nordfertil Research Lab Stockholm, Department of Women's and Children's Health, Karolinska Institutet and University Hospital Karolinska Institutet, Stockholm, Sweden; ^2^Paediatric Endocrinology Unit, Department of Women's and Children's Health, Karolinska Institutet and University Hospital Karolinska Institutet, Stockholm, Sweden; ^3^College of Medicine and Health Sciences, Sultan Qaboos University, Muscat, Oman; ^4^Department of Clinical Science, Intervention and Technology (CLINTEC), Karolinska Institutet and University Hospital Karolinska Institutet, Stockholm, Sweden

## Abstract

Human embryonic stem (hES) cells represent an important tool to study early cell development. The previously described use of human recombinant laminin (LN) 521 represented a step forward in generating clinically safe culture conditions. To test the short-term effect of LN521 on cultured hES cells, five male hES cell lines were cultured on human foreskin fibroblasts (hFFs), Matrigel, LN521, and LN121 and characterized by qPCR, immunofluorescence analysis, as well as their potential for three-germ layer differentiation. Variations in gene expression related to pluripotency, stemness, and testicular cells at different passages and culture conditions were evaluated by qPCR. All cell lines expressed pluripotency markers at protein and RNA level and were able to differentiate into cell types of the three germ layers after being cultured on LN521 for nine passages. Reduction in variation of pluripotency marker expression could be observed after culturing the cells on LN521 for nine passages. hES cells cultured on LN521 exhibited less differentiation, faster cell growth, and attachment when compared to hES cells cultured on LN121 or Matrigel. Our results indicate a positive effect of LN521 in stabilizing pluripotency gene expression and might be the first step towards more controllable and robust culture conditions for hES cells.

## 1. Introduction

Human embryonic stem (hES) cells, together with induced pluripotent stem cells, provide a unique platform to study molecular and cellular mechanisms in humans. Although hES cells are isolated at a very early stage of development, between five to eight days after fertilization [1, 2] and have the potential to give rise to the three germ layers, different cell lines seem to vary in their capacity to proliferate and to differentiate. They exhibit diverse expression profiles and seem to prefer various differentiation pathways [3, 4]. In addition to these cell line-specific profiles, the differentiation potential has been shown to be method- and even laboratory-dependent [5, 6]. Thus, new strategies involving the employment of well-defined and controlled culture conditions are needed to establish robust hES cell differentiation protocols.

Conventionally, hES cells are cocultured on feeder cells [7], but the use of hES cells in future personalized medicine requires xeno-free and ideally even feeder-free culture conditions [8–10]. Such xeno- and feeder-free culture conditions are needed to avoid immunogenicity, microbial or viral contamination, and batch-to-batch variability of the culture matrices used [11]. In the first attempts to create a feeder-free culture system, Matrigel which is a protein mixture derived from mouse sarcoma cells, containing laminin (LN) 111, type IV collagen, perlecan, and nidogen, as well as several unknown components and growth factors, was used [12]. To a large degree, these unknown components and the batch-to-batch variability of Matrigel complicate comparability between hES cell experiments [13].

In order to avoid variability, well-defined culture conditions, involving, for example, purified matrix proteins such as LN521, combined with xeno-free media, have been designed to further increase the reliability and reproducibility of various differentiation protocols used [8, 14–16]. Recently used for directive differentiation of human pluripotent stem cells into several cell types, for example, hepatic cells, retinal pigment epithelial cells, and dopaminergic neurons [17–19], these culture conditions can be seen as a major step towards the application of pluripotent stem cell lines in personalized medicine.

In addition to the already mentioned advantages of using LN521, a reduction of DNA damage in hES cells cultured on LN521, compared with cultures on mouse feeder cells, has been reported as soon as after a single passage [20]. However, evaluation of gene expression profiles involving several hES cell lines generated on feeder cells and transferred onto LN521 with special focus on the differences during the first passages and the effects on pluripotency gene expression has not been performed.

Hence, in the present study, we aimed to investigate the short-term effects of LN521 on the expression of different genes, including the expression of common pluripotency genes in feeder cell-derived hES cell lines after being transferred to and cultured on LN521 for up to nine passages.

## 2. Material and Methods

### 2.1. Ethics

Ethics approval for the derivation and differentiation of the hES cell lines used in this study was obtained from the Regional Ethics Committee in Stockholm (Dnr. 454/02).

### 2.2. Cell Lines

Human ES cell lines HS360, HS364, HS380, HS401, and HS420, all karyotype 46,XY, were derived by mechanical isolation of the inner cell masses of six-day-old embryos. Karyotypes were confirmed to be normal 46,XY in all five cell lines after 17–40 passages on human foreskin fibroblasts (hFFs). Before culturing the hES cells on LN521- (LN521-02, human recombinant laminin 521, #80104, Biolamina, Sweden), LN121- (LN121-02, human recombinant laminin 121, test samples from Biolamina, Sweden), and Matrigel- (Corning Matrigel hESC-qualified matrix, 354277, Corning, Bedford, MA, US) coated plates, hES cells were cultured on mitotically inactivated hFFs (CRL-2429, ATCC, Manassas, VA, USA) in knock-out (KO) DMEM medium (KO-DMEM, 10829018, Invitrogen, Life Technologies, Paisley, Scotland) containing 20% KO serum replacement (KO-SR, 10828028, Invitrogen), 0.5% penicillin-streptomycin (15140122, Invitrogen), 2 mM L-GlutaMAX (35050038, Invitrogen), 1% nonessential amino acids (11140035, Invitrogen), 0.5 mM 2-mercaptoethanol (31350010, Invitrogen), and recombinant human fibroblast growth factor 2 at 8 ng/ml (FGF2, 234-FSE/CF, R&D Systems, Minneapolis, USA). In addition to hES cell cultures on hFFs in DMEM, as described above, hES cells were cultured on inactivated hFFs (CRL-2429, ATCC, Manassas, VA, USA) in NutriStem medium (NutriStem hESC XF, 05-100-1A, Biological Industries Israel Beit Haemek Ltd., Israel) at 37°C in 5% CO_2,_ with daily medium change. The cell colonies were passaged mechanically at four- to six-day intervals.

To enable hES cell culture on LN521- and LN121-coated plates, LN521 and LN121 were slowly thawed at 4°C for one to two hours and diluted at a 1 : 10 ratio with DPBS containing CaCl_2_ and MgCl_2_ (DPBS^++^) to a final concentration of 10 *μ*g/ml. The plates were then coated with LN521 or LN121 at 5 *μ*g/cm^2^ overnight at 4°C followed by one hour at 37°C or for two hours at 37°C according to the manufacturer's instructions. The hES cells were cultured in NutriStem medium (NutriStem hESC XF, 05-100-1A, Biological Industries Israel Beit Haemek Ltd., Israel) at 37°C and with 5% CO_2_, with daily medium change.

To perform hES cell culture on Matrigel-coated plates, Matrigel was thawed on ice at 4°C overnight. Plates were incubated with Matrigel diluted 1 : 84 in NutriStem at 4°C overnight or at room temperature for 1 hour.

The cells were passaged enzymatically using TrypLE select (12563011, Invitrogen) every five to seven days. After three to four passages, the cells were expanded to collect material for analysis.

### 2.3. Spontaneous Differentiation: Embryoid Body Formation

At passage nine, after reaching confluence on LN521-coated plates, hES cells were allowed to form embryoid bodies (EBs) in 24-well ultralow attachment plates (#3473; Corning) for 14 days in NutriStem without growth factors (#06-5100-01-1A; Biological Industries). The EBs were then either plated on LN521-coated culture plates or chamber slides (#354104, Corning) for an additional 14 days. After a total of 28 days, differentiated cells were either used for gene expression analysis or fixed with 4% paraformaldehyde for immunocytofluorescence analysis.

### 2.4. RNA Isolation and cDNA Amplification

Human ES cells were harvested using enzymatic digestion with TrypLE select. RNA isolation was performed using an RNeasy kit (#74104, Qiagen, Germany) according to the manufacturer's protocol and treated with DNase (RNase-free DNase Set, 79254, Qiagen). cDNA was synthesized using an iScript cDNA synthesis kit (#170-8891, Bio-Rad), following the manufacturer's protocol.

### 2.5. Gene Expression Analysis

#### 2.5.1. Taqman Low-Density Assay

To compare the five hES cell lines, the expression of 96 genes was analysed using Taqman low-density (TLD) array cards (4385344, Applied Biosystems, Carlsbad, CA 92008, USA), designed by the International Stem Cell Initiative, according to the manufacturer's protocol. Briefly, the TLD array cards were preloaded with 96 TaqMan probes which were run at the same time. Included in the 96 genes were markers of undifferentiated stem cells, pluripotency maintenance, stemness and differentiation, and six internal controls. The data was analysed using RQ-Manager 1.2 software. All the hES cell lines were run in three biological replicates.

#### 2.5.2. Quantitative Real-Time PCR

Quantitative PCR was performed using TaqMan Universal PCR Master Mix (#4369016, Applied Biosystem) with TaqMan probes (Applied Biosystem) according to the manufacturer's protocol (Supplementary Table 1). GAPDH was used as an internal control.

### 2.6. DNA Isolation and Karyotyping

After harvesting the hES cells with TrypLE, DNA was isolated using a DNeasy Blood and Tissue kit (69504, Qiagen) according to the manufacture's manual. Photospectrometry was used to control DNA concentration and purity. Karyotyping was performed at the Finnish Functional Genomics Centre, Turku, Finland, on the basis of BACs-on-Beads technology, using bead-bound bacterial artificial chromosome probes, which allows the analysis to be performed with only a low amount of DNA isolated from the whole cell pool, as described previously [21].

### 2.7. Immunocytofluorescence Analysis

For immunocytofluorescence and immunofluorescence analysis, the cells were fixed with 4% paraformaldehyde for 15 minutes, washed three times with 1× phosphate-buffered saline (PBS) and permeabilized with 0.3% Triton X-100 (#108643, Merck, Germany) in PBS for ten minutes. After blocking for one hour with 5% donkey serum (125558, Jackson ImmunoResearch), 1% bovine serum albumin (BSA; 001-000-162, Jackson ImmunoResearch) and 0.1% Tween 20 (#655205, Merck) in 1× PBS at room temperature, the cells were incubated with primary antibodies in antibody solution (1% donkey serum, 0.1% BSA, and 0.1% Tween20 in PBS) at 4°C overnight (Supplementary Table 2). On the next day, the cells were washed three times with 1× PBS and incubated with secondary antibodies (donkey anti-mouse Alexa Fluor 488 conjugated (715-546-150, Jackson ImmunoResearch) or donkey anti-rabbit Cy3 conjugated (711-166-152, Jackson ImmunoResearch)) in an antibody solution for one hour at room temperature in the dark and afterwards counterstained with DAPI at 1 *μ*g/ml (PureBlu DAPI, 135-1303, BioRad) for 15 minutes. The slides were visualized and images were taken using a confocal microscope (LSM700, Zeiss, Germany). A detailed description of the primary antibodies used in the study can be found in Supplementary Table 2.

### 2.8. Statistical Analysis

Gene expression (qPCR) was calculated as the mean ± standard deviation (SD) of triplicates run for each cell line. Expression was normalized against the expression of *GAPDH* as a housekeeping gene. One-way ANOVA was applied to compare the differences between the five cell lines (SigmaPlot 11.0, Sysat Software Inc., California, USA). Following the Shapiro–Wilk test for normality, pair-wise multiple comparisons were performed by using the Holm–Sidak procedure or Tukey's test (SigmaPlot 11.0, Sysat Software Inc.). A difference was considered to be statistically significant if the *p* value was <0.05.

TLD array data was normalized using *GAPDH* as a housekeeping gene control, and undetected genes were removed. To separate and compare gene expression profiles of cell lines, the dCT means of replicates were plotted in a heat map, separating cell lines using hierarchical clustering. Moreover, common patterns of expression were investigated via intersection of genes with high expression, arbitrarily defined as exhibiting dCT < 5, as well as low expression (dCT > 15).

### 2.9. Coefficient of Variation Calculation

The coefficient of variation (CV) of each gene was obtained by calculating the ratio between the SD and the mean value of all the cell lines together. Based on the CV value, variation was classified into four groups: (I) up to 10%, (II) 11–25%, (III) 26–50%, and (IV) more than 50%.

## 3. Results

### 3.1. Heterogeneous Gene Expression Levels of hES Cell Lines Transferred onto LN521

**Figure 1:**
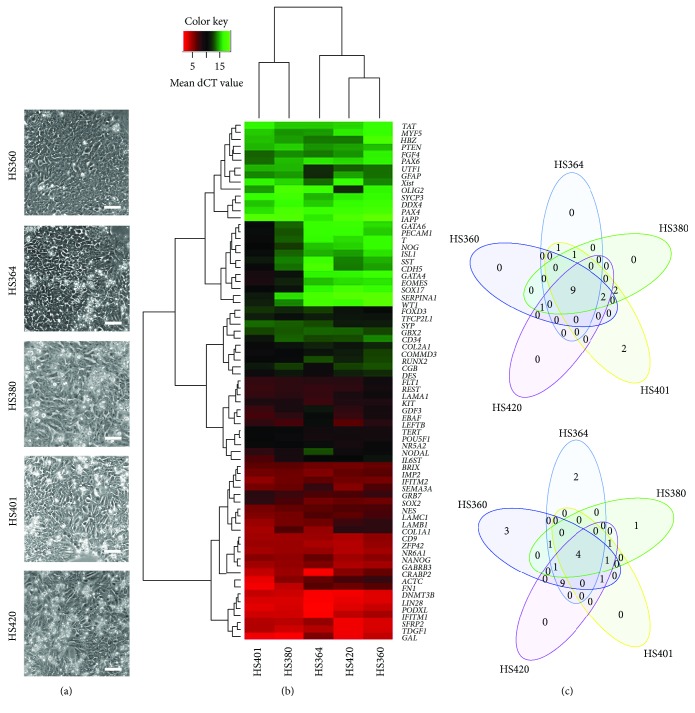
hES cells derived on hFFs and cultured to p4 on LN521. All cell lines (HS360, HS364, HS380, HS401, and HS420) cultured on LN521 exhibited typical hES cell morphology at p4 (a). The hierarchical clusters of gene expression levels for all the cell lines analysed by TLD arrays show a heterogeneous expression profile specific for every cell line. Red colour represents high gene expression (low dCT value) and green colour represents low gene expression (high dCT value) (b). As shown by the Venn diagram, all five cell lines exhibited higher expression of nine stemness-related genes and lower expression of four genes related to differentiation (c).

To examine the behaviour of hES cells derived on hFFs during the first passages in feeder-free culture conditions, we grew five male hES cell lines (HS360, HS364, HS380, HS401, and HS420) for four passages (p4) on LN521-coated plates. The colonies formed monolayers and continued to grow without signs of accelerated differentiation (Figure 1(a)).

To confirm the pluripotent nature of the five hES cell lines, gene expression analysis of 96 genes using TLD arrays revealed similar but not identical expression profiles (Figure 1(b) and Supplementary Table 3). At p4, all five cell lines exhibited enhanced expression of nine genes (*ZFP42*, *TDGF1*, *SFRP2*, *PODXL*, *NR6A1*, *CD9*, *DNMT3B*, *IFITM1*, and *LIN28*), which are all related to stemness, and reduced expression of four genes (*TAT*, *PAX4*, *IAPP*, and *DDX4*), which are related to differentiation (Figure 1(c)). Karyotypes were confirmed to be 46,XY in all five cell lines after p4 on LN521 (data not shown).

### 3.2. Stable Pluripotency of hES Cell Lines Derived on hFFs and Transferred onto LN521

**Figure 2:**
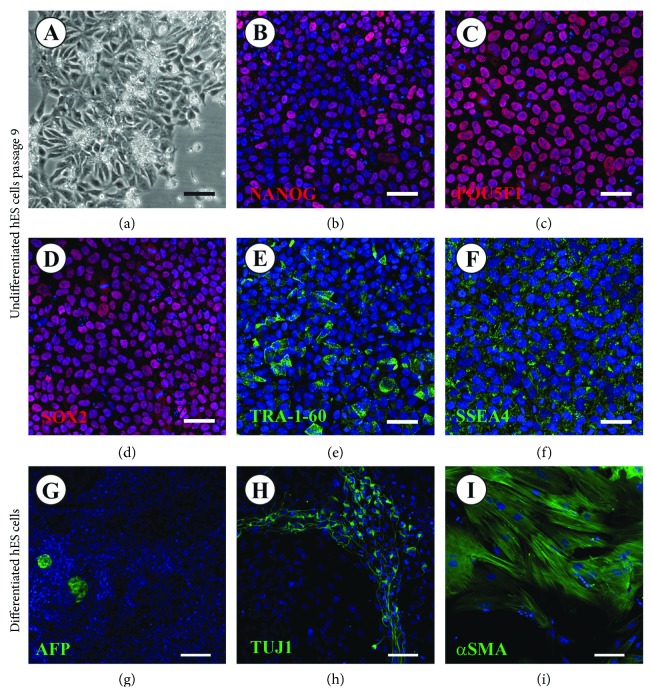
hES cells derived on hFFs and cultured to p9 on LN521. All five cell lines transferred from hFFs onto LN521 exhibited normal hES cell morphology (a). Immunofluorescence analysis of NANOG (b), POU5F1 (c), SOX2 (d), SSEA4 (e), and TRA-1-60 (f) expression, shown for HS380 as a representative cell line, revealed the presence of pluripotency markers after p9 on LN521 (red staining: NANOG, POU5F1, and SOX2; green staining: SSEA4 and TRA-1-60; and blue staining: DAPI). Scale bars: 50 *μ*m. Immunofluorescence analysis of the expression of differentiation markers for endoderm (AFP) (g), ectoderm (TUJ1) (h), and mesoderm (alpha SMA) (i), shown in HS380 as a representative cell line, demonstrates the potential of hES cells cultured on LN521 to differentiate spontaneously into the three germ layers (green staining: AFP, alpha SMA and TUJ1; blue staining: DAPI). Scale bars: 50 *μ*m.

The pluripotency status and potential influence of the culture method per se on the five hES cell lines cultured on LN521 were tested after prolonged culture (nine passages). The morphology of cultured cells was not changed compared with cells cultured up to p4 (Figures 1(a) and 2(a)). All five cell lines expressed a set of five commonly known pluripotency genes at the protein level. Immunofluorescence staining revealed nuclear expression of NANOG, SOX2, and POU5F1 and cytoplasmic expression of SSEA4 and TRA-1-60 (Figures 2(b)–2(f) and Supplementary Figure 1). Quantitative analysis of the pluripotency genes expressed in the nuclei, in all five cell lines, revealed no significant differences between the cell lines in the expression of NANOG (98% ± 2%), POU5F1 (98% ± 1%), and SOX2 (99% ± 1%) (Supplementary Table 4).

After p9 on LN521, the ability of the cells to differentiate spontaneously into the three germ layers (ecto-, meso-, and endoderm) *in vitro* by the formation of EBs was tested. All hES cell lines expressed markers of ectoderm, mesoderm, and endoderm after 28 days of spontaneous differentiation (Figures 2(g)–2(i) and Supplementary Figure 2). Cells differentiating to endoderm revealed AFP secretion. Cells exhibiting expression of TUJ1 in their cytoskeletons demonstrated cells of ectodermal origin. Cells positive for alpha SMA protein expression were identified as mesodermal cells. qPCR analysis confirmed the RNA expression of endoderm-, mesoderm-, and ectoderm- as well as trophoblast-related genes. *GATA6*, *AFP*, and *DDX4* were used as endodermal, *KDR* and *ACTC1* as mesodermal, *NEUROD1* and *PAX6* as ectodermal markers, and *KRT7* as a trophoblast marker (Supplementary Figure 3).

### 3.3. Similarity in Expression Levels of Genes Related to Pluripotency

**Figure 3:**
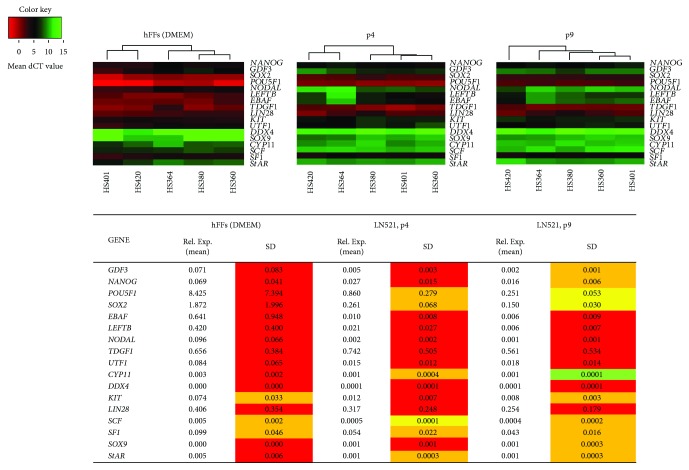
Homogenization of the expression of pluripotency genes and genes related to male germ cells and somatic gonadal cells after prolonged culture of hES cells on LN521 presented as heat maps for dCT values on hFFs at p4 and LN521 at p4 and p9. Red colour represents high gene expression (low dCT value) and green colour represents low gene expression (high dCT value); undetected (low/no expression) samples was given a dCT value of 15. (Below) a table of the mean and SD values of the five cell lines showing the relative expression values of pluripotency genes and genes related to stemness and male gonadal cells to *GAPDH*. The coefficient of variation is calculated as SD/mean and the variations among the lines are classified into four groups: less than 10% (green), 11% to 25% (yellow), 26%–50% (orange), and more than 50% (red).

In order to investigate the effect of utilizing LN521 as a matrix for hES cell culture, we analysed the expression of 17 genes by qPCR. Since LN521 has been shown to be present in decellularized matrix of the human adult testis [22], we included markers of male gonadal cells in the analysis (Figure 3 and Supplementary Table 5). When comparing the gene expression profile of cells cultured on hFFs in DMEM with cells cultured on hFFs in NutriStem, a similar expression pattern was observed (Supplementary Table 6).

However, when comparing the gene expression profiles of hES cultured on hFFs and LN521 at p4, among the 17 genes investigated, 14 genes showed more than 50% gene expression variation on hFFs compared to 11 genes showing more than 50% gene expression variation on LN521. While at p9 of LN521 culture, only seven genes showed more than 50% variation (Figure 3).

Homogenization of pluripotency gene expression in all five cell lines was observed when comparing expression variation between p4 and p9. At p9, two pluripotency genes (*NANOG* and *GDF3*) showed a variation of less than 50% (34% and 28%, resp.) from more than 50% at p4 (54% and 66%, resp.). *POU5F1* and *SOX2* showed a reduction in variation from less than 50% (32% and 26%, resp.) at p4 to less than 25% (21% and 20%, resp.) at p9.

To explore the impact of culturing hES cells using LN521 matrix on other genes important for early cell development, we analysed RNA expression of genes related to stemness and germ and somatic cell markers at p4 and p9. *NODAL*, *LEFTB*, *EBAF*, *TDGF1*, *UTF1*, and *LIN28* showed a variation of more than 50% for both p4 and p9. However, variation in the expression of *LEFTB*, *UTF1*, and *LIN28* was reduced at p9 compared to p4 (Figure 3).

Genes related to gonadal cells showed a different pattern of variation in their expression between p4 and p9. *DDX4* and *StAR* showed no variation in their expression, while *KIT*, *CYP11*, *SF1*, and *SOX9* showed less variation at p9. In contrast, SCF showed more variation in its expression at p9 (Figure 3).

To investigate the difference between LN521 and other matrices used for hES cell culture, we cultured two hES cell lines (HS380 and HS360) on LN521, LN121, and Matrigel for four passages (Supplementary Figures 4 and 5) and one hES cell line (HS380) for nine passages on LN521 and LN121 and seven passages for Matrigel (Supplementary Figure 4). We observed a higher incidence of differentiation in cells cultured on Matrigel and LN121 compared to those cultured on LN521. In addition, hES cells were attaching and growing at a lower rate on LN121 compared to LN521. The differences between LN521, LN121, and Matrigel became more pronounced with prolonged culture (p7 to p9) compared to p4 (Supplementary Figure 4).

## 4. Discussion

Since the first isolation of hES cells, in 1998, the derivation and establishment of clinically safe hES cell lines have proven to be challenging [23, 24]. Incompletely defined culture conditions and the use of xenogenic materials for hES cell culture and expansion have limited interpretation and understanding of the mechanisms controlling self-renewal, pluripotency, and differentiation characteristics. Thus, the possibility to use human pluripotent stem cells in personalized medicine is limited [13]. The establishment of more defined culture conditions, for example, through the use of synthetic matrices would result in better-controlled conditions.

It was suggested that hES cells express four different isoforms of laminin, LN521, LN121, LN511, and LN111, since they express alpha 1, alpha 5, beta 1, beta 2, and gamma 1 laminin chains [9]. In addition, alpha 5 chain laminins are found in the inner cell mass (ICM) of the mouse blastocyst which indicates that alpha 5 containing laminin isoforms are part of stem cell niches [25]. Therefore, we thought that LN521 might be an optimal matrix not only for cultures of newly derived pluripotent stem cells but also for hES cells previously derived and cultured on hFFs. A successful and robust adaptation of hES cells derived on hFFs and cultured in feeder- and xeno-free conditions using LN521 would allow that more and well-characterized hES cell lines can be used to establish defined differentiation protocols.

In the present study, the expression profiles of five XY hES cell lines, which were derived on hFFs, were analysed in regard to their pluripotency after short-term culture on LN521. We cultured hES cell lines (HS360, HS364, HS380, HS401, and HS420) on LN521 to p4 and p9. All five hES cell lines grew in a monolayer on LN521-coated wells, as previously described [9]. A confluent layer was observed within five to seven days without showing accelerated signs of differentiation. After being transferred from hFFs to LN521 and cultured to p4, all five cell lines showed a normal 46,XY karyotype. These results are in line with those of previous studies showing the feasibility of LN521 as a matrix for hES cell cultures in xeno-free and chemically defined culture systems [8]. However, the long-term impact on their genomic stability needs to be examined further to confirm the results of our short-term cultures.

Despite the overall pluripotent nature of the five cell lines, variation in expression of genes for pluripotency and differentiation was observed, indicating cell line-specific expression patterns. However, when comparing the expression of pluripotency genes (*SOX2*, *NANOG*, *POU5F1*, and *GDF3*) between the five cell lines cultured on hFFs and LN521 (p4), a homogenized expression profile was observed for hES cells cultured on LN521. Moreover, with prolonged culture (p9) on LN521, the gene expression variation was reduced further when compared to p4 on LN521. This homogenization between different cell lines cultured on LN521 indicates more predictable and robust culture conditions for hES cells, which are useful requirements for the establishment of directed and cell line-independent differentiation protocols.

To assess if this homogenization effect occurs due to the culture on LN521 or the change to NutriStem, cell culture medium, we cultured four cell lines (HS360, HS364, HS380, and HS401) for four passages on hFFs in two different media, in DMEM with supplements and in NutriStem. Our results showed a similar picture of variation in the expression of pluripotency genes and genes related to stemness in both cell culture media. This indicates that change to NutriStem medium when culturing the cells on LN521 had no obvious effect.

The expression of LN521 in the decellularized matrix obtained from human testis has been described earlier [22]. Therefore, we examined potential effects of LN521 on gene expression levels in early germ and somatic cells, by including markers usually expressed in human foetal male gonads. We investigated the expression of genes related to the specification and appearance of early stages of primordial germ cells in humans (*KIT, UTF1, LIN28,* and *DDX4*) and genes related to gonadal somatic cells (*CYP11, SOX9, SCF, StAR*, and *SF1*) as well as genes expressed in undifferentiated hES cells and related to the NODAL pathway (*EBAF, LEFTB, NODAL*, and *TDGF1*), which plays a major role in early cell fate, including germ-cell specification and endoderm specification as well as maintenance of the pluripotent status of stem cells [26]. Our results showed that LN521 had no effect on the expression levels of genes related to early gonadal cells, for example, for germ-cell specification and differentiation.

In addition, we observed that LN521 provides a more suitable matrix for hES cell attachment, growth, and self-renewal compared to Matrigel and LN121. A detailed study of the effect of long-term culture of hES on LN521 and LN121 compared with the short-term culture described in this study will be needed in the future to confirm the stability of gene expression profiles of hES cells cultured on LN521. The difference in the alpha chain between LN521 and LN121 as culture matrices may explain the diverse effects on cell attachment and gene expression levels on the hES cells. However, as mentioned above, the effect of this difference and its impact needs a thorough study for better understanding.

## 5. Conclusions

Our results demonstrate that LN521 supports hES cell growth and has a positive effect on maintaining the cells in a normal balanced pluripotent state. Culture on LN521 leads to homogenization of the gene-expression profiles of the different hES cell lines derived on hFFs, used in this study. Similar expression profiles of different hES cell lines might result in more predictable and therefore controllable behaviour of these cells in various differentiation protocols, which are required for the possible use of these cell lines in the field of future regenerative medicine.

## Additional Points

*Highlights*. Laminin 521 provides suitable culture conditions for adaptation to feeder- and xeno-free conditions of hES cells derived and cultured on feeder cells. Laminin 521 has a positive effect in homogenizing pluripotent gene expression profiles between hES cell lines cultured for up to nine passages. Laminin 521 has no obvious effect on the expression of genes related to male germ cells and somatic gonadal cells, when used as a matrix for hES cell cultures.

## Supplementary Materials

Supplementary 1 Figure 1: Immunofluorescence analysis of NANOG, POU5F1, SOX2, SSEA4 and TRA-1-60 expression, shown for HS360, HS364, HS401 and HS420 cells, revealed the presence of pluripotency markers after p9 on LN521 (red staining: NANOG, POU5F1 and SOX2; green staining: SSEA4 and TRA-1-60; blue staining: DAPI). Scale bars: 50 *μ*m.

Supplementary 2 Figure 2: Immunofluorescence analysis of protein expression in the three germ layers (endoderm (AFP), ectoderm (TUJ1) and mesoderm (alpha SMA)) was positive for all markers in HS360, HS364, HS401 and HS420 cells cultured on LN521 after spontaneous differentiation. Green staining: AFP, alpha SMA and TUJ1; blue staining: DAPI. Scale bars: 50 *μ*m.

Supplementary 3 Figure 3: QPCR analysis of genes related to differentiation for all cell lines on LN521: mesodermal markers KDR and ACTC1; ectodermal markers PAX6 and NEUROD1; endodermal markers GATA6, AFP and DDX4; trophoblast marker KRT7. Gene expression relative to that of *GAPDH* is shown before (grey bars) and after spontaneous differentiation (red bars). Abbreviations: ND: not detected.

Supplementary 4 Figure 4: Comparison of RNA expression of pluripotency genes, genes related to stemness and genes expressed in the male gonadal cells, for hES cells cultured on three different matrices (LN121, LN521 and Matrigel). The differences in the gene expression for HS380 at p4 on LN121, LN521 and Matrigel, and at p9 on LN121 and LN521, and at p7 on Matrigel are presented as heatmap for dCT value normalized to *GAPDH*. Red colour represents high gene expression (low dCT value) and green colour represents low gene expression (high dCT value). Undetected (low/no expression) samples was given a dCT value of 13. Abbreviation: SD: standard deviation; dCT: delta cycle threshold.

Supplementary 5 Figure 5: Comparison of RNA expression of pluripotency genes, genes related to stemness and genes expressed in the male gonadal cells, for hES cells cultured on three different matrices (LN121, LN521 and Matrigel). The differences in the genes expression for HS360 at p4 on LN121, LN521 and matrigel are presented as heatmap for dCT value, normalized to *GAPDH*. Red colour represents high gene expression (low dCT value) and green colour represents low gene expression (high dCT value). Undetected (low/no expression) samples was given a dCT value of 13. Abbreviations: dCT: delta cycle threshold.

Supplementary 6 Table 1: Detailed information for the TaqMan primers (Applied Biosystems, ThermoFisher Scientific) used to analyse the RNA expression of genes related to pluripotency, stemness and differentiation, and genes expressed in male gonadal cells. Table 2: Detailed information for the primary antibodies used to analyse the protein expression of pluripotent, mesodermal, endodermal and ectodermal cells as well as for IgGs used as negative controls.

Supplementary 7 Table 3: Data set of the mean dCT value (±SD) of gene expression for cell lines cultured on LN521 (four passages), normalized to *GAPDH*. Mean dCT values (±SD) for all genes included in the TLD array. Abbreviations: dCT: delta cycle threshold; SD: standard deviation; TLDA: taqman low density array.

Supplementary 8 Table 4: Quantitative analysis of protein expression of pluripotency genes (NANOG, POU5F1 and SOX2) in five hES lines cultured on LN521 for 9 passages. No significant difference between the lines in the expression of all three genes. Counting is presented as mean (±SD) of the percentage of the number of positive cells compared to total number of cells. Abbreviation: SD: standard deviation.

Supplementary 9 Table 5: Data set of the mean dCT values (±SD) of gene expression of cell lines cultured on hFFs, LN521 p4 and LN521 P9, normalized to *GAPDH*. Mean dCT values (±SD) for all genes was analysed with qPCR. Abbreviations: dCT: delta cycle threshold; SD: standard deviation; hFFs: human foreskin fibroblasts.

Supplementary 10 Table 6: hES cells cultured on feeder cells in DMEM at p3 and p4 with supplements and NutriStem at p4 show no difference in expression variation of pluripotency genes and genes related to stemness. The mean values (±SD) for the five cell lines showing the relative expression to *GAPDH* of pluripotency genes and genes related to stemness and genes expressed in male gonadal cells. The coefficient of variation is calculated as SD/mean and the variations among the lines are classified into four groups: less than 10% (green), 11% to 25% (yellow), 26%–50% (orange) and more than 50% (red). Abbreviation: SD: standard deviation; Rel. Exp.: relative expression.
