# Supplementary material for: Laminin 521 Stabilizes the Pluripotency Expression Pattern of Human Embryonic Stem Cells Initially Derived on Feeder Cells
Source: Stem Cells Int. 2018 Feb 18;2018:7127042. doi: 10.1155/2018/7127042 (PMC5835285; doi:10.1155/2018/7127042)
Supplement: Supplementary 6 — Table 1: Detailed information for the TaqMan primers (Applied Biosystems, ThermoFisher Scientific) used to analyse the RNA expression of genes related to pluripotency, stemness and differentiation, and genes expressed in male gonadal cells. Table 2: Detailed information for the primary antibodies used to analyse the protein expression of pluripotent, mesodermal, endodermal and ectodermal cells as well as for IgGs used as negative controls. [file 7127042.f6.docx]

Supplementary Table 1:

| **Gene** | **Gene name** | **Assay ID** |
| --- | --- | --- |
| *POU5F1* | POU class 5 homeobox 1 | Hs03005111-g1 |
| *GDF3* | growth differentiation factor 3 | Hs00220998-m1 |
| *SOX2* | SRY-box 2 | Hs01053049 |
| *NANOG* | Nanog homeobox | Hs02387400-g1 |
| *KDR* | kinase insert domain receptor | Hs00176676-m1 |
| *ACTC1* | actin, alpha, cardiac muscle 1 | Hs01109515-m1 |
| *GATA6* | GATA-binding protein 6 | Hs00232018-m1 |
| *NEUROD1* | neuronal differentiation 1 | Hs00159598-m1 |
| *AFP* | alpha fetoprotein | Hs00173490-m1 |
| *PAX6* | paired box 6 | Hs01088112-m1 |
| *KRT7* | keratin 7 | Hs00818825-m1 |
| *DDX4* | DEAD-box helicase 4 | Hs00987133-m1 |
| *SOX9* | SRY-box 9 | Hs01001343-g1 |
| *CYP11* | cytochrome P450 family 11 subfamily A member 1 | Hs00167984-m1 |
| *SCF* | KIT ligand | Hs00241497-m1 |
| *SF1* | splicing factor 1 | Hs00190309-m1 |
| *StAR* | steroidogenic acute regulatory protein | Hs00986559-g1 |
| *NODAL* | nodal growth-differentiation factor | Hs00415443-m1 |
| *EBAF* | left-right-determination factor 2 | Hs00745761-s1 |
| *LEFTB* | left-right-determination factor 1 | Hs00764128-s1 |
| *LIN28* | lin-28 homolog A | Hs00702808-s1 |
| *KIT* | KIT proto-oncogene receptor tyrosine kinase | Hs00174029-m1 |
| *UTF1* | undifferentiated embryonic cell-transcription factor 1 | Hs00864535-s1 |
| *TDGF1* | teratocarcinoma-derived growth factor 1 | Hs02339498-gH |
| *GAPDH* | Human GAPD (GAPDH) endogenous control | 4333764F |

Supplementary Table 2:

| Protein | Host | Antibody  concentration | Antibody  dilution | Catalogue number |
| --- | --- | --- | --- | --- |
| TRA-1-60 | mouse | 2 mg/ml | 1:200 | ab16288 |
| SSEA4 | mouse | 1 mg/ml | 1:40 | ab16287 |
| OCT4 (POU5F1) | rabbit | 0.2 mg/ml | 1:150 | ab19857 |
| NANOG | rabbit | 0.2 mg/ml | 1:50 | ab21624 |
| SOX2 | rabbit | 1 mg/ml | 1:500 | ab97959 |
| AFP | mouse | 1 mg/ml | 1:200 | ab3980 |
| Tubulin beta 3 (TUJ1) | mouse | 1 mg/ml | 1:500 | BioSite, 801201 |
| α-SM1 (alpha SMA) | mouse | 200 µg/ml | 1:500 | sc-130616 |
| Mouse IgG | mouse | 200 µg/0.5ml |  | sc-2025 |
| Rabbit IgG | rabbit | 0.2 mg/ml |  | ab27478 |
