# Supplementary material for: Laminin 521 Stabilizes the Pluripotency Expression Pattern of Human Embryonic Stem Cells Initially Derived on Feeder Cells
Source: Stem Cells Int. 2018 Feb 18;2018:7127042. doi: 10.1155/2018/7127042 (PMC5835285; doi:10.1155/2018/7127042)
Supplement: Supplementary 7 — Table 3: Data set of the mean dCT value (±SD) of gene expression for cell lines cultured on LN521 (four passages), normalized to GAPDH. Mean dCT values (±SD) for all genes included in the TLD array. Abbreviations: dCT: delta cycle threshold; SD: standard deviation; TLDA: taqman low density array. [file 7127042.f7.docx]

Supplementary Table 3:

|  | **hESC lines** | | | | |
| --- | --- | --- | --- | --- | --- |
| **Genes** | **HS360** | **HS364** | **HS380** | **HS401** | **HS420** |
| *ACTC* | 7.2(0.2) | 6.0(0.1) | 5.0(3.8) | 2.5(4.1) | 6.8(0.5) |
| *BRIX* | 5.9(0.2) | 5.7(0.2) | 6.3(0.1) | 6.2(0.4) | 5.6(0.4) |
| *CD34* | 13.9(0.1) | 12.6(0.7) | 13.2(2.2) | 10.9(1.5) | 12.7(0.5) |
| *CD9* | 4.8(0.1) | 4.5(0.1) | 4.4(0.3) | 4.3(0.2) | 4.0(0.6) |
| *CDH5* | 14.5(0.4) | 17.6(0.2) | 13.4(5.6) | 9.8(4.6) | 13.7(0.9) |
| *CGB* | 12.9(0.2) | 9.3(0.0) | 12.4(1.4) | 11.2(0.9) | 12.5(0.5) |
| *COL1A1* | 7.6(0.2) | 4.6(0.2) | 6.6(3.7) | 4.2(2.5) | 7.7(0.5) |
| *COL2A1* | 11.7(0.2) | 11.6(0.1) | 10.8(1.3) | 9.7(1.3) | 11(0.4) |
| *COMMD3* | 12.5(0.1) | 10.8(0.1) | 9.9(1.5) | 9.5(1.8) | 11.3(0.2) |
| *CRABP2* | 5.9(0.1) | 1.9(0.3) | 4.4(0.9) | 3.5(0.9) | 5.1(0.1) |
| *DDX4* | 16.8(2.6) | 15.3(0.1) | 16.4(0.7) | 15.2(0.0) | 16.4(1.5) |
| *DES* | 11.9(0.2) | 9.2(0.4) | 11.0(2.2) | 9.3(1.6) | 11.5(0.0) |
| *DNMT3B* | 2.7(0.3) | 2.0(0.2) | 3.1(0.8) | 3.4(1.2) | 2.1(0.3) |
| *EBAF* | 8.6(0.2) | 10.4(0.1) | 7.5(0.7) | 6.9(1.2) | 7.2(1.7) |
| *EOMES* | 15.8(0.2) | 15(1.1) | 11.6(5.3) | 8.3(5.9) | 15.6(1.4) |
| *FGF4* | 16.0(0.4) | 13.7(0.1) | 14.1(0.7) | 13.2(0.9) | 14.3(0.4) |
| *FLT1* | 9.1(0.4) | 7.8(0.2) | 7.5(0.2) | 7.4(0.5) | 7.8(0.7) |
| *FN1* | 6.5(0.2) | 6.4(0.1) | 3.5(3) | 2.1(4.1) | 5.8(1.3) |
| *FOXD3* | 10.3(0.2) | 12.5(0.2) | 11.7(0.5) | 12.1(1.4) | 10.8(0.6) |
| *GABRB3* | 4.3(0.2) | 5.3(0.1) | 4.7(0.1) | 4.3(0.4) | 3.9(0.6) |
| *GAL* | 3.4(0.1) | 5.3(0.1) | 1.8(0.5) | 2.2(1.4) | 2.3(1.3) |
| *GATA4* | 16.7(0.6) | 16.0(1.5) | 10.8(8.6) | 8.5(6.8) | 16.1(0.7) |
| *GATA6* | 15.6(0.6) | 16.6(0.4) | 13.3(6.8) | 10.2(8.9) | 16.7(0.4) |
| *GBX2* | 12.7(0.6) | 13.3(0.2) | 13(2.3) | 12.6(3.1) | 13.2(1.1) |
| *GDF3* | 8.7(0.1) | 11(0.2) | 8.4(1.3) | 7.2(0.7) | 7.5(0.3) |
| *GFAP* | 13.5(0.2) | 11.8(0.2) | 14.4(0.1) | 13.9(0.2) | 14.5(0.9) |
| *GRB7* | 7.2(0.0) | 6.7(0.1) | 7.2(0.7) | 7.8(0.8) | 6.7(0.1) |
| *HBZ* | 17.5(1.2) | 13.7(0.5) | 14.4(0.3) | 15(0.8) | 13.7(0.2) |
| *IAPP* | 19.0(0.0) | 15.8(0.3) | 17.9(0.0) | 18.1(0.0) | 17.8(0.0) |
| *IFITM1* | 4.3(0.0) | 1.7(0.2) | 3.1(0.5) | 3.1(1.1) | 3.5(0.8) |
| *IFITM2* | 5.5(0.1) | 6.2(0.1) | 5.7(1.1) | 4.8(1) | 5.6(0.3) |
| *IL6ST* | 10.8(0.0) | 10(0.1) | 8.5(3) | 6.7(3.2) | 10.1(0.8) |
| *IMP2* | 6.3(0.1) | 5.2(0.1) | 5.7(0.1) | 5.6(0.5) | 6.0(0.8) |
| *ISL1* | 15.7(0.4) | 14.8(0.5) | 13.3(4.1) | 10.4(3.7) | 14.4(0.1) |
| *KIT* | 8.1(0.2) | 7.1(0.0) | 8.3(0.5) | 8.2(0.5) | 8.1(0.1) |
| *LAMA1* | 9.2(0.1) | 7.3(0.3) | 7.7(0.9) | 7.4(1.2) | 8.3(0.7) |
| *LAMB1* | 7.6(0.2) | 5.7(0.1) | 5.5(1.8) | 4.5(2.2) | 7.1(1.0) |
| *LAMC1* | 6.9(0.1) | 6.5(0.1) | 5.5(0.8) | 5.2(1.3) | 6.3(0.9) |
| *LEFTB* | 7.8(0.1) | 10.5(0.1) | 6.9(1.2) | 7.5(0.8) | 6.3(2.3) |
| *LIN28* | 2.8(0.1) | 1.8(0.1) | 2.8(0.4) | 2.9(0.3) | 2.4(0.2) |
| *MYF5* | 16.5(1.1) | 14.4(0.7) | 14.2(0.5) | 15.2(0.4) | 15.9(0.5) |
| *NANOG* | 4.9(0.1) | 5.9(0.1) | 4.7(0.3) | 5.0(0.5) | 4.3(0.4) |
| *NES* | 6.8(0.1) | 6.2(0.2) | 6.0(0.9) | 5.5(0.8) | 6.6(0.4) |
| *NODAL* | 9.6(0.2) | 12.7(0.1) | 8.6(2.5) | 7.6(2.5) | 9.1(1.5) |
| *NOG* | 16.3(0.4) | 15.1(1.3) | 12.3(4.1) | 10.2(4.1) | 15.3(0.3) |
| *NR5A2* | 9.6(0.1) | 10.4(0.2) | 9.9(0.2) | 9.7(0.7) | 8.6(0.2) |
| *NR6A1* | 4.2(0.1) | 4.6(0.3) | 4.5(0.2) | 4.2(0.3) | 3.8(0.3) |
| *OLIG2* | 16.1(0.7) | 16.7(0.0) | 17.6(0.2) | 14.3(3.1) | 11.9(0.2) |
| *PAX4* | 17.3(0.8) | 16.8(0.7) | 17.6(1.5) | 15.8(0.8) | 16.9(0.0) |
| *PAX6* | 16.1(1.5) | 15.8(1.3) | 15.0(1.9) | 13.4(1.6) | 15.5(1.7) |
| *PECAM1* | 15.3(0.5) | 16.5(0.6) | 12.9(4.9) | 10.4(5.7) | 16.5(1.1) |
| *PODXL* | 3.2(0.2) | 1.2(0.0) | 2.9(0.2) | 2.6(0.1) | 2.7(0.3) |
| *POU5F1* | 8.8(0) | 8.8(0.0) | 9.5(0.7) | 10.2(1.1) | 8.8(1.6) |
| *PTEN* | 15(0.2) | 14.2(0.6) | 15.3(1.1) | 14.8(0.7) | 14.5(0.5) |
| *REST* | 8.7(0.1) | 7.3(0.1) | 7.6(0.5) | 7.1(0.7) | 7.5(0.2) |
| *RUNX2* | 12.6(0.2) | 12.7(0.5) | 10.4(1.1) | 10.3(1.8) | 11.6(0.7) |
| *SEMA3A* | 6.7(0.1) | 7.4(0.2) | 5.8(0.2) | 5.6(0.4) | 5.1(1.1) |
| *SERPINA1* | 18.6(1.2) | 17.4(0.8) | 15.6(5.1) | 11.2(6.3) | 18.5(2.3) |
| *SFRP2* | 2.9(0.1) | 4.6(0) | 3.7(0.4) | 3.8(0.9) | 2.2(0.4) |
| *SOX17* | 16.4(0.1) | 16.1(1.2) | 10.8(7.5) | 10.1(8.5) | 17.3(1.4) |
| *SOX2* | 5.7(0.1) | 5.6(0.1) | 6.7(1.5) | 7.6(1.9) | 5.9(0.8) |
| *SST* | 14.7(0.7) | 16.8(0.8) | 13.9(3.8) | 11.2(2.9) | 13.8(0.6) |
| *SYCP3* | 16.3(0.4) | 14.8(0.5) | 17.0(0.4) | 16.3(1.1) | 15.8(1.2) |
| *SYP* | 11.3(0.2) | 12.7(0.1) | 13.2(1.6) | 13.3(2.4) | 11.2(0.6) |
| *T* | 16.3(0.0) | 16.3(1.7) | 13.2(6.7) | 9.7(6.2) | 15.6(1.0) |
| *TAT* | 16.5(0.3) | 15.2(0.5) | 15.2(0.9) | 16.2(1.3) | 15.4(0.7) |
| *TDGF1* | 3.0(0.1) | 3.8(0.2) | 3.1(1.3) | 3.9(1.3) | 2.3(0.0) |
| *TERT* | 8.7(0.2) | 8.9(0.1) | 9.7(1.0) | 10.3(1.5) | 8.8(0.4) |
| *TFCP2L1* | 10.8(0.3) | 11.4(0.3) | 10.6(0.5) | 11.4(0.5) | 10.9(0.4) |
| *UTF1* | 13.5(0.5) | 12(0.7) | 14.9(0.8) | 14.8(1.2) | 13.1(1.3) |
| *WT1* | 18.5(0.8) | 17.1(0.1) | 13.9(4.3) | 11.5(0.0) | 16.6(0.8) |
| *Xist* | 13.9(0.6) | 13.4(0.2) | 15.4(0.4) | 16.1(0.0) | 16.4(0.8) |
| *ZFP42* | 4.6(0.2) | 4.0(0.2) | 4.5(0.2) | 4.5(0.3) | 3.8(0.3) |
