# Supplementary material for: Laminin 521 Stabilizes the Pluripotency Expression Pattern of Human Embryonic Stem Cells Initially Derived on Feeder Cells
Source: Stem Cells Int. 2018 Feb 18;2018:7127042. doi: 10.1155/2018/7127042 (PMC5835285; doi:10.1155/2018/7127042)
Supplement: Supplementary 8 — Table 4: Quantitative analysis of protein expression of pluripotency genes (NANOG, POU5F1 and SOX2) in five hES lines cultured on LN521 for 9 passages. No significant difference between the lines in the expression of all three genes. Counting is presented as mean (±SD) of the percentage of the number of positive cells compared to total number of cells. Abbreviation: SD: standard deviation. [file 7127042.f8.docx]

Supplementary Table 4:

| **Cell line** | **NANOG**  [Mean±SD (%)] | **POU5F1**  [Mean±SD (%)] | **SOX2**  [Mean±SD (%)] |
| --- | --- | --- | --- |
| HS360 | 97 ± 1 | 98 ± 2 | 99 ± 2 |
| HS364 | 97 ± 3 | 99 ± 1 | 99 ± 1 |
| HS380 | 98 ± 2 | 98 ± 1 | 98 ± 1 |
| HS401 | 98 ± 1 | 98 ± 2 | 98 ± 1 |
| HS420 | 98 ± 1 | 98 ± 1 | 99 ± 1 |
