# Supplementary material for: Laminin 521 Stabilizes the Pluripotency Expression Pattern of Human Embryonic Stem Cells Initially Derived on Feeder Cells
Source: Stem Cells Int. 2018 Feb 18;2018:7127042. doi: 10.1155/2018/7127042 (PMC5835285; doi:10.1155/2018/7127042)
Supplement: Supplementary 9 — Table 5: Data set of the mean dCT values (±SD) of gene expression of cell lines cultured on hFFs, LN521 p4 and LN521 P9, normalized to GAPDH. Mean dCT values (±SD) for all genes was analysed with qPCR. Abbreviations: dCT: delta cycle threshold; SD: standard deviation; hFFs: human foreskin fibroblasts. [file 7127042.f9.docx]

Supplementary Table 5:

| **Mean dCT values ±SD for all genes analysed with qPCR from cells cultured on feeders** | | | | | |
| --- | --- | --- | --- | --- | --- |
| **Genes** | **HS360** | **HS364** | **HS380** | **HS401** | **HS420** |
| *NANOG* | 4.4(0.5) | 5.5(0.3) | 4.1(0.3) | 3.4(0.3) | 3.1(0.5) |
| *GDF3* | 5.3(0.9) | 5.4(0.1) | 6.8(0.7) | 2.3(0.5) | 3.8(0.5) |
| *SOX2* | -0.3(0.6) | 0.4(0.6) | -0.1(0.5) | -2.1(0.9) | -0.4(0.5) |
| *POU5F1* | -2.7(0.7) | -1.3(0.1) | -0.8(0.6) | -4.4(0.3) | -3.4(0.4) |
| *NODAL* | 4.6(0.9) | 5.3(0.1) | 3.9(0.7) | 2.5(0.1) | 2.7(0.4) |
| *LEFTB* | 2.4(1.5) | 0.3(2.3) | 1.4(1.8) | 1.7(0.3) | 0.4(0.4) |
| *EBAF* | 2.4(0.8) | 1.4(0.7) | 0.7(2.4) | 0.9(0.5) | 1.0(0.4) |
| *TDGF1* | 1.2(0.8) | 3.0(0.2) | 0.1(0.6) | 0.0(0.1) | 0.5(0.4) |
| *LIN28* | 2.8(0.2) | 2.3(0.6) | 2.8(0.6) | 1.5(0.6) | 1.8(0.6) |
| *KIT* | 3.4(0.7) | 4.0(0.5) | 3.7(0.6) | 3.8(0.4) | 4.5(0.1) |
| *UTF1* | 4.6(0.8) | 4.7(0.3) | 4.2(0.5) | 2.5(0.5) | 3.5(0.3) |
| *DDX4* | 15.0(0.0) | 15.0(0.0) | 15.0(0.0) | 15.0(0.0) | 11.9(0.0) |
| *SOX9* | 15.0(0.0) | 11.1(0.2) | 15.0(0.0) | 15.0(0.0) | 11.1(0.4) |
| *CYP11* | 8.7(0.5) | 10.9(0.5) | 8.6(0.2) | 7.5(0.3) | 8.9(0.5) |
| *SCF* | 7.9(1.0) | 7.7(0.0) | 8.5(1.0) | 7.1(0.4) | 7.4(0.4) |
| *SF1* | 3.7(0.9) | 4.1(0.3) | 3.8(0.4) | 2.5(0.2) | 3.4(0.3) |
| *StAR* | 9.0(0.6) | 9.5(0.3) | 8.6(0.2) | 6.2(0.8) | 7.7(0.6) |

**Mean dCT values ±SD for all genes analysed with qPCR at P4**

| **Genes** | **HS360** | **HS364** | **HS380** | **HS401** | **HS420** |
| --- | --- | --- | --- | --- | --- |
| *NANOG* | 4.6(0.5) | 6.6(0.5) | 4.8(0.2) | 5.3(0.2) | 5.9(0.1) |
| *GDF3* | 7.5(0.8) | 8.5(0.4) | 7.2(0.4) | 7.0(0.3) | 10.1(0.4) |
| *SOX2* | 2.1(0.1) | 1.8(0.4) | 2.6(0.2) | 1.9(0.2) | 1.6(0.1) |
| *POU5F1* | -0.2(0.2) | 0.8(0.3) | 0.0(0.2) | 0.2(0.6) | 0.6(0.1) |
| *NODAL* | 8.1(0.5) | 12.5(0.3) | 8.5(0.1) | 8.9(0.0) | 11.7(0.6) |
| *LEFTB* | 4.2(1.1) | 11.9(1.0) | 6.9(0.3) | 5.0(0.2) | 7.8(0.2) |
| *EBAF* | 5.8(0.6) | 10.9(0.7) | 6.8(0.2) | 6.1(0.1) | 7.9(0.2) |
| *TDGF1* | 0.6(0.4) | 1.5(0.6) | 0.3(0.3) | -0.6(0.3) | 1.8(0.3) |
| *LIN28* | 2.1(0.1) | 2.2(0.2) | 3.7(0.0) | 1.9(0.1) | 0.4(0.0) |
| *KIT* | 6.9(0.1) | 5.3(0.2) | 7.5(0.1) | 6.7(0.1) | 6.2(0.0) |
| *UTF1* | 8.4(0.4) | 5.1(0.5) | 7.5(0.2) | 6.4(0.3) | 5.3(0.1) |
| *DDX4* | 14.7(0.8) | 12.7(0.9) | 14.6(0.5) | 13.0(0.5) | 13.1(0.5) |
| *SOX9* | 11.2(0.2) | 9.4(0.4) | 10.9(0.1) | 10.3(0.2) | 9.0(0.2) |
| *CYP11* | 10.5(0.5) | 10.4(0.1) | 11.4(0.3) | 9.6(0.3) | 9.7(0.2) |
| *SCF* | 11.1(0.1) | 11.5(0.0) | 11.2(0.2) | 10.5(0.0) | 11.3(0.4) |
| *SF1* | 4.3(0.5) | 5.1(0.2) | 4.4(0.4) | 4.4(0.3) | 3.5(0.0) |
| *StAR* | 10.6(0.3) | 9.7(0.3) | 10.1(0.5) | 9.9(0.2) | 10.5(0.1) |
| **Mean dCT values ±SD for all genes analysed with qPCR at P9** | | | | | |
| **Genes** | **HS360** | **HS364** | **HS380** | **HS401** | **HS420** |
| *NANOG* | 6.0(0.2) | 6.2(0.0) | 5.6(0.2) | 6.9(0.4) | 5.5(0.4) |
| *GDF3* | 9.3(0.4) | 9.2(0.1) | 7.7(0.2) | 9.4(0.5) | 9.5(0.2) |
| *SOX2* | 3.0(0.1) | 2.5(0.2) | 2.7(0.3) | 3.1(0.2) | 2.5(0.1) |
| *POU5F1* | 1.9(0.1) | 2.2(0.1) | 1.7(0.1) | 2.4(0.3) | 1.9(0.2) |
| *NODAL* | 11.3(0.5) | 11.3(0.5) | 10.1(0.5) | 10.8(1.5) | 8.1(0.3) |
| *LEFTB* | 7.7(0.8) | 10.1(0.4) | 8.8(0.8) | 7.7(1.5) | 6.4(0.5) |
| *EBAF* | 8.4(0.8) | 10.1(0.5) | 8.9(0.6) | 9.0(1.5) | 6.1(0.7) |
| *TDGF1* | 1.0(0.6) | 0.8(1.2) | 0.9(0.2) | 0.9(1.8) | 1.9(0.4) |
| *LIN28* | 2.5(0.4) | 2.7(0.2) | 3.8(0.4) | 1.8(0.6) | 1.0(0.2) |
| *KIT* | 7.0(0.3) | 6.3(0.3) | 7.0(0.2) | 7.5(0.1) | 7.6(0.1) |
| *UTF1* | 7.3(0.1) | 4.6(0.3) | 6.7(0.2) | 6.6(0.4) | 5.5(0.2) |
| *DDX4* | 13.7(0.6) | 12.5(0.9) | 15.1(0.3) | 14.3(1.4) | 14.5(1.0) |
| *SOX9* | 11.3(0.6) | 10.2(0.1) | 10.7(0.3) | 10.9(0.1) | 9.9(0.6) |
| *CYP11* | 10.8(0.5) | 10.6(0.1) | 11.6(0.3) | 11.2(0.1) | 10.9(0.3) |
| *SCF* | 11.8(0.4) | 11.6(0.2) | 10.4(0.2) | 12.1(0.3) | 10.6(0.3) |
| *SF1* | 4.9(0.6) | 5.1(0.3) | 4.5(0.3) | 4.9(0.1) | 3.9(0.1) |
| *StAR* | 9.9(0.3) | 10.1(0.1) | 9.8(0.2) | 10.5(0.2) | 11.6(0.2) |
