# Supplementary material for: Laminin 521 Stabilizes the Pluripotency Expression Pattern of Human Embryonic Stem Cells Initially Derived on Feeder Cells
Source: Stem Cells Int. 2018 Feb 18;2018:7127042. doi: 10.1155/2018/7127042 (PMC5835285; doi:10.1155/2018/7127042)
Supplement: Supplementary 10 — Table 6: hES cells cultured on feeder cells in DMEM at p3 and p4 with supplements and NutriStem at p4 show no difference in expression variation of pluripotency genes and genes related to stemness. The mean values (±SD) for the five cell lines showing the relative expression to GAPDH of pluripotency genes and genes related to stemness and genes expressed in male gonadal cells. The coefficient of variation is calculated as SD/mean and the variations among the lines are classified into four groups: less than 10% (green), 11% to 25% (yellow), 26%–50% (orange) and more than 50% (red). Abbreviation: SD: standard deviation; Rel. Exp.: relative expression. [file 7127042.f10.docx]

| **GENE** | **Feeders, DMEM** | | **Feeders, NutriStem** | |
| --- | --- | --- | --- | --- |
|  | **Rel.Exp. (mean)** | **SD** | **Rel.Exp. (mean)** | **SD** |
| ***GDF3*** | 0,071 | **0,083** | 0,0849 | **0,042** |
| ***NANOG*** | 0,069 | **0,041** | 0,0607 | **0,034** |
| ***POU5F1*** | 8,425 | **7,394** | 6,6958 | **3,849** |
| ***SOX2*** | 1,872 | **1,996** | 1,7745 | **1,624** |
| ***EBAF*** | 0,641 | **0,948** | 2,4139 | **2,216** |
| ***LEFTB*** | 0,420 | **0,400** | 0,8463 | **0,607** |
| ***NODAL*** | 0,096 | **0,066** | 0,1380 | **0,095** |
| ***TDGF1*** | 0,656 | **0,384** | 0,6909 | **0,394** |
| ***UTF1*** | 0,084 | **0,065** | 0,0798 | **0,047** |
| ***CYP11*** | 0,003 | **0,002** | 0,0027 | **0,000** |
| ***DDX4*** | 0,000 | **0,000** | 0,0000 | **0,000** |
| ***KIT*** | 0,074 | **0,033** | 0,1105 | **0,062** |
| ***LIN28*** | 0,406 | **0,354** | 0,1986 | **0,063** |
| ***SCF*** | 0,005 | **0,002** | 0,0059 | **0,006** |
| ***SF1*** | 0,099 | **0,046** | 0,0815 | **0,026** |
| ***SOX9*** | 0,000 | **0,000** | 0,0003 | **0,000** |
| ***StAR*** | 0,005 | **0,006** | 0,0028 | **0,001** |

Supplementary Table 6:

Green: CV up to 10%

Yellow: CV above 10 up to 25%

Orange: CV above 25 up to 50%

Red: CV above 50%
